# Shesha, a WhatsApp Chatbot for Linking Household Contacts to Tuberculosis Treatment or Preventive Therapy in South Africa: Design and Development

**DOI:** 10.2196/71793

**Published:** 2025-11-13

**Authors:** Don Lawrence Mudzengi, Thobani Ntshiqa, Yohhei Hamada, Felex Ndebele, Thapelo Mpanza, Bridget Kyobutungi, Candice Williams, Meghan Kennealy, Molebogeng Rangaka, Kavindhran Velen, Salome Charalambous

**Affiliations:** 1 The Aurum Institute, 3 Wrench Road, Isando, Johannesburg, Gauteng, 1600, South Africa, 27 83 369 7946; 2 School of Public Health, University of the Witwatersrand, Johannesburg, South Africa; 3 Institute of Global Health, University College London, London, United Kingdom; 4 Reach Digital Health, Johannesburg, South Africa

**Keywords:** contact tracing, design thinking, development, Health Belief Model, linkage to care, mHealth, patient engagement, tuberculosis, tuberculosis preventive treatment, universal TB testing, WhatsApp chatbot, Shesha

## Abstract

**Background:**

Literature on the development of mobile health (mHealth) tools for public health interventions is scarce. This scarcity creates a knowledge gap, and new tools may repeat the mistakes of past implementations.

**Objective:**

In this paper, we describe the development of Shesha, a WhatsApp-based chatbot designed to facilitate linkage to care for household contacts of people being treated for tuberculosis (TB). Shesha facilitates linkage by providing TB test results, TB preventive treatment (TPT) information, nudges, reminders, and personalized support. We developed Shesha to address the human resource capacity challenges posed by South Africa’s new universal TB testing and TPT policies.

**Method:**

We applied a design thinking framework with 7 phases: empathize, discover, define, prototype, build and launch, improve, and evaluate. The process started with gathering insights from TB contact tracing studies and consulting with global and local experts to address the challenges of universal TB testing and TPT. Based on these findings, we defined the core functionalities of Shesha and incorporated them in the Health Belief Model to encourage health-seeking behavior. In collaboration with the developers, we developed the WhatsApp-based chatbot. We selected WhatsApp for its wide accessibility and user-friendliness.

**Results:**

We successfully developed and launched the Shesha in September 2023, with implementation expected to continue until March 2025. Early user acceptance revealed that users generally valued the information provided on the tool regarding TB and TPT; however, they required ongoing engagement to link to care. Ongoing evaluations, guided by the Reach Effectiveness Adoption Implementation Maintenance (RE-AIM) framework, will assess the tool’s impact on reducing community health worker workloads and improving linkage to care.

**Conclusions:**

Documenting the development of mHealth technologies is crucial for guiding future projects and improving health interventions. In our study, frameworks like design thinking and the Health Belief Model aligned Shesha with user needs and programmatic goals. Comprehensive documentation may help assess the chatbot’s performance and guide future improvements, supporting scalability and efficiency in mHealth interventions across public health settings.

## Introduction

South Africa’s tuberculosis (TB) control program is increasingly turning to mobile health (mHealth) tools to support service delivery across the care cascade. Yet, most of these tools remain poorly evaluated and underdocumented [1,2], limiting opportunities for replication and adaptation at scale. In 2019, the South African TB Think Tank, a government-led consortium of TB experts, found that none of the 10 mHealth tools they reviewed met the government standards for design simplicity and robust evaluation [3], and our 2024 systematic review had similar findings, including the absence of documenting how the tools were developed [4]. Franck and colleagues further argue that innovation can only advance if successes and failures are openly documented; as they put it, “to err is evolution” [5]. Although recent studies from Argentina [6], the United Kingdom [5], Tanzania [6], and Uganda [7] have begun setting a precedent, evidence for TB-focused tools in South Africa remains limited.

The urgency to close this documentation gap is evident in South Africa’s TB program, responsible for controlling one of the largest epidemics globally, with 270,000 new people infected with TB and 25,000 deaths annually [8]. The country runs an extensive contact tracing program to find TB among household contacts, but it relies on a labor-intensive cascade of *finding the contacts, screening them, collecting specimens, delivering results and reminders, and linking them to care* [9-13]. This approach is characterized by repetitive household visits by community health workers (CHWs), a model already proven to be suboptimal [9,12,13]. In South Africa, as seen in other African contexts, this model also continues to face challenges such as contacts not being traced, high dropout rates along the cascade, poor clinic attendance, and generally exorbitant costs for its delivery [13,14]. In 2023, South Africa introduced a new policy that expanded and further strained the delivery of TB contact tracing done by CHWs. The policy requires all household contacts to undergo microbiological testing and makes every contact who tests negative now eligible for tuberculosis preventive treatment (TPT) [15,16]. Under the previous regime, microbiological testing was limited to symptomatic contacts, and TPT eligibility applied only to children under 5 years and people living with HIV. This new policy targets 250,000 TPT initiations in contacts by 2026, from only 17,000 [16], a 15-fold increase which without innovation cannot be realistically met by CHWs. The enormity of this burden thus requires digital solutions whose evidence remains limited in the country.

We responded to the demands of the new policy and the need to support CHWs in contact tracing by developing Shesha. This mHealth tool was a WhatsApp (WhatsApp LLC, Menlo Park, CA, USA) chatbot, which enabled household contacts to undertake repetitive activities usually performed by CHWs, such as receiving test results, engaging with motivational messages, receiving clinic visit reminders, and confirming linkage to care. By reallocating these tasks, Shesha could free up time for CHWs to focus on reaching more households and meeting the expanded obligations under the new policy. At the time we developed Shesha, we were not aware of any tool in South Africa that allowed household contacts to assume this role. In this paper, we document Shesha’s development and share the design choices and processes behind it, to provide an evidence base that others working in TB mHealth can use or adapt.

## Methods

### Setting and Context

We embedded the Shesha development in the intervention arm of the Community and Universal Testing for Tuberculosis among Contacts (CUT-TB) trial, a European and Developing Countries Clinical Trials Partnership (EDCTP)–funded cluster-randomized trial [17]. The primary objective of this 2-phase trial was to evaluate the effectiveness of universal testing compared with standard TB screening for detecting TB among household and community contacts.

We implemented in the Ekurhuleni district, one of the 5 districts in the Gauteng Province in South Africa. Ekurhuleni is one of the most populous districts in the Gauteng province, with a growing population estimated to be just over 4 million residents (27% of Gauteng's population) spread over 1975 km^²^. Most residents in Ekurhuleni are multilingual [18,19]; however, about 34% residents are native IsiZulu speakers, a language also intelligible to an additional 8% who speak isiXhosa, and a much smaller fraction who speak isiNdebele and siSwati [18]. Ekurhuleni is highly urbanized and serves as an industrial hub, but it also experiences high levels of poverty, as evidenced by numerous informal settlements [20]. These settlements are characterized by a high burden of TB and other infectious diseases. It accounted for 25% of Gauteng's 31,704 TB diagnoses reported in 2024 [21] and the district has the highest HIV prevalence among pregnant women in Gauteng province at 28.5%, according to a recent antenatal sentinel survey [22]. Internet connectivity and smartphone ownership have grown rapidly. According to Statistics South Africa, about 82% of the population now has internet access, with 75.7% in Gauteng accessing the internet using mobile phones which can serve as a proxy for smartphone ownership [23]. Although we implemented the Shesha in Ekurhuleni, we did not develop it as a district-specific tool; instead, its deployment in Ekurhuleni was primarily operational due to the location of the CUT-TB trial.

### Study Population

Our target population for the Shesha chatbot consisted of household contacts of TB patients in the Ekurhuleni District. With the expanded contact tracing policy in South Africa, all contacts underwent microbiological testing. Our study staff collected sputum from all contacts who could provide, regardless of whether they exhibited symptoms or not. Those who tested positive were referred for TB treatment; those testing negative were directed to start TPT. The latter group posed a challenge because most were generally healthy, with low perceived risk, minimal interest in visiting clinics, and limited motivation to start treatment. The study staff enrolled eligible and consenting household contacts into Shesha on the day they collected the sputum. Both TPT and the use of the Shesha tool were entirely new to many of these contacts. This low-risk perception and health-seeking behavior are common among household contacts. A South African TB prevalence survey found that over 60% of symptomatic household members delayed seeking care, often only doing so after prompting [24].

### Design Thinking Framework for Developing Shesha

**Figure 1.**
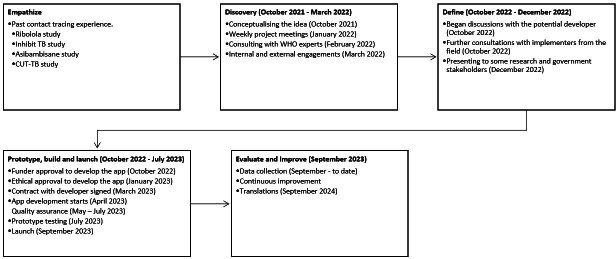
Design thinking framework. CUT-TB, Community and Universal Testing for Tuberculosis among Contacts; TB, tuberculosis; WHO, World Health Organization.

We used a design thinking framework for the Shesha development process (see Figure 1). This framework has 7 phases: empathize, discover, define, prototype, build and launch, improve, and evaluate [25]. Some studies on case finding have used these design thinking frameworks as structured methodologies for solving complex problems and creating user-centric solutions [26,27]. These frameworks ensure that user needs are addressed while achieving public health goals and incorporate participatory consultation with stakeholders. The following sections describe the activities conducted under each phase of the design thinking framework.

### Empathize

The empathize phase involved data collection to understand user needs and ensure that solutions we create, such as Shesha, align with their challenges. However, in our case, the situation we faced with the targeted users made conventional approaches infeasible, such as interviewing them before development. As we outlined in the study population section, the household contacts that we aimed to support with Shesha had no prior exposure to TPT, and many were unaware of their risk or that they were eligible for any preventive care. Within this context, therefore, we determined that engaging them upfront could have generated speculation rather than any actionable input into Shesha’s design. Despite the potential weaknesses of our approach and the circumstances that were identified, it still recognized the importance of capturing user perspectives. Immediately after launching Shesha, an early user satisfaction survey was conducted for all contacts enrolled up to March 2024. While not equivalent to a textbook predeployment empathize phase, this offered authentic user perspectives at the earliest feasible opportunity in our process.

Our judgment of the circumstances limiting us from getting early insights led us to leverage existing evidence and our extensive experience as researchers in contact tracing. This approach also aligns with other established guidance that behavior change interventions can be informed by theory, prior evidence, and formative research [28]. We therefore drew on insights from our previous contact tracing studies, which include Ribolola [29], Inhibit-TB [13], Asibambisane [19], and the current CUT-TB trial [17]. These studies collectively identified the central challenges of TB contact tracing: poor linkage to care, high attrition, overstretched CHWs, and the pressures created by universal testing. They also highlighted specific needs and gaps, such as the value of community-based care (Ribolola), the importance of reliable scheduling and repeated follow-up (Inhibit-TB), the limits of CHW capacity and variability in workloads (Asibambisane), and the staffing shortages and policy demands associated with universal testing (CUT-TB). We designed and developed, incorporating this understanding of the contact tracing program. Shesha thus featured mobile result sharing with contacts, educational content on TB and TPT via WhatsApp, automated follow-ups, passive clinic scheduling support, and treatment initiation verification.

### Discover

#### Overview

During the discovery phase, we gathered insights into both the challenges and potential solutions for universal TB testing and universal TPT. We engaged global and local experts through a participatory process to evaluate whether Shesha was an appropriate response to these challenges and to validate its relevance in addressing the identified issues, while also securing their buy-in.

#### Internal Discussions

In late 2021, before engaging external stakeholders, we held internal discussions between Aurum and University College London researchers to refine the concept of Shesha. These discussions were part of the weekly operational meetings for the CUT-TB project. We brainstormed ideas on how to create a system that would enable linkage to care for household contacts of people with TB after the introduction of the universal testing and TPT policy for all household contacts. We acknowledged that while universal testing could take place during household visits, maintaining consistent follow-up and ensuring successful linkage to care posed a challenge too complex for CHWs to manage alone, which led to the idea of developing Shesha.

#### External Engagements

At the beginning of 2022, after brainstorming and finalizing a draft of our concept, we began approaching external experts. We first held a meeting in February 2022 with representatives from the World Health Organization to gather their input on how to approach the overall change in South Africa’s TPT policy. We also presented our concept for designing a tool to facilitate linkage to care for these contacts under the universal testing policy. Their main suggestion to our idea was to add options that would allow enrolling community contacts if feasible. Community contacts are people who do not live in the same household as the person with TB but have regular contact with them.

From this consultation, we further developed our concept within the internal team and started ad hoc discussions with potential developers. In March 2023, an organization was approached to explore using an existing tool called CommCare but did not materialize, and then in August 2022, discussions were held with the developers whom we finally selected.

For the external stakeholders, we consulted public health officials from the health departments and TB/HIV program implementers, mainly from the Global Fund to Fight AIDS, Tuberculosis and Malaria and HIV the President’s Emergency Plan for AIDS Relief (PEPFAR). The local stakeholders generally agreed that Shesha had the potential to address some of the challenges in the contact tracing cascade, especially after the introduction of a universal policy, by automating follow-up processes and alleviating the burden on CHWs. They also recommended a rigorous evaluation of the tool.

### Define

#### Theoretical Reasoning Behind Shesha

We developed Shesha as a “patient-facing” WhatsApp chatbot to streamline the linkage to care for contacts of people with TB. It allowed the contacts to interact with it by sending and receiving messages in automated prompts that mimic key follow-up tasks which CHWs usually perform. These are primarily administrative, repetitive tasks that include sending TB results, confirming contacts read results, visiting the clinic, starting treatment, supporting adherence, and confirming completion of treatment. By automating these tasks, Shesha potentially reduces the need for manual follow-up by CHWs, thereby alleviating their workload from repeated home visits.

We based our design on the Health Belief Model (HBM) to capture underlying drivers of health-related behavior change [30]. The use of HBM in our study helped predict and promote health behaviors by addressing attitudes, beliefs, and perceptions about TB, its treatment, and prevention. It allowed us to tailor messages to the user’s psychological and emotional motivators, drawing them closer to the desired action of starting treatment after receiving results. The HBM is well suited for our target group, the household contacts of people with TB, who do not inherently perceive themselves as susceptible to the disease. Unlike alternative theories such as the Theory of Reasoned Action and the Theory of Planned Behavior, which assume that individuals already recognize a need for action and rely on rationality in making decisions, the HBM uses individual constructs for people who have not made a decision.

**Figure 2.**
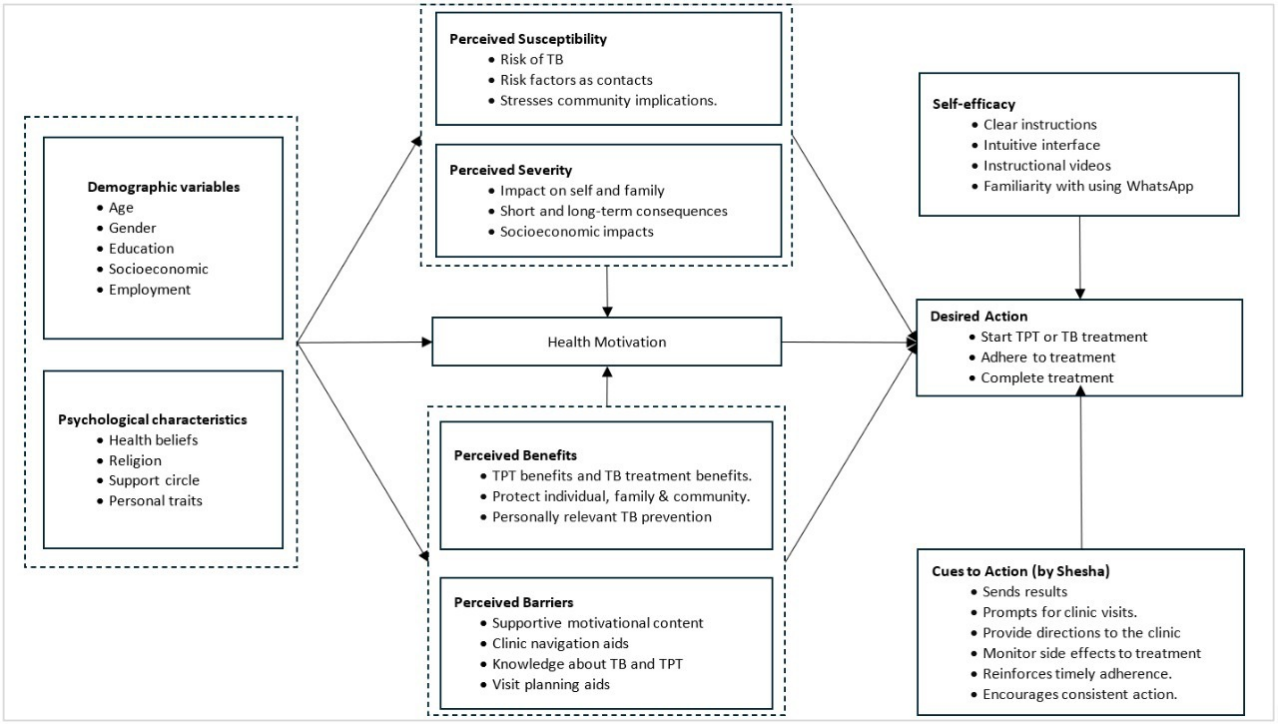
Health Belief Model. TB: tuberculosis; TPT: tuberculosis preventive treatment.

Shesha operationalizes all HBM domains to guide users through the TB contact tracing and linkage cascade. Household contacts have a low *perceived susceptibility* to TB risk, as well as *perceived severity* if they are infected. Most are unaware of TPT; hence, their perception of its benefits is poor. We, however, provide information to reduce obstacles that might hinder their access to care and address their perceived barriers to care. Shesha continuously engages users by providing cues to action, sending reminders, prompting clinic visits, encouraging medication adherence, and offering health messaging. To strengthen their self-efficacy in staying on the linkage journey, Shesha delivers precise, easily understandable instructions on how to visit the clinic, what to plan, and an intuitive WhatsApp interface with which most are familiar. This approach aimed to encourage users to engage with Shesha messages, visit the clinic, and start and adhere to treatment, reducing attrition along the contact tracing cascade. Figure 2 details how each domain of the HBM has been applied.

#### Engaging With Developers

We identified the developers whom we considered an ideal development partner for Shesha due to their extensive experience with mHealth tools, such as the COVID Alert and TB-Alert apps [31]. Their familiarity with the South African health landscape and local infrastructure also made them particularly suitable for this project. We negotiated contracts to address the scope of the work, design, and key legal issues, mainly how to handle intellectual property emanating from the Shesha design, publication rights. We used feedback from stakeholder engagements to refine the project scope. In early 2023, the funder approved the proposal to develop Shesha. After this, we began translating the findings from the empathize phase and discovery stages to define the chatbot’s key functionalities and platform choices. We also outlined roles and responsibilities for the development team between the two organizations; the research provided the concepts with a wireframe showing how Shesha should function, and developers translated that into a chatbot prototype. We continued with regular meetings with the developers throughout this journey up to the end of the implementation.

#### Choosing the Platform to Develop Shesha

Our budget permitted us to choose only 1 platform, so we selected WhatsApp. However, our initial presentations to local stakeholders and the developers were based on an Unstructured Supplementary Service Data (USSD) system. Local stakeholders preferred WhatsApp because it was more likely to be sustained by the government compared to alternatives like SMS or USSD, which entail high long-term provider costs. A similar mHealth tool, “MomConnect,” cost approximately US $60,000 monthly in 2018, even after being subsidized by the network provider, and it was based on USSD and SMS [32]. MomConnect now incorporates WhatsApp. WhatsApp is also now widely used in South Africa, where over 45 million people use the internet, and at least 94% of them actively use WhatsApp [33,34].

The developers also recommended WhatsApp based on precedent; it had been widely used in South Africa for COVID-19 self-screening. Some of the key points that informed our decision-making process are outlined in Table 1. Our design requirements included an intuitive interface, an effective notification system, 2-way interaction with sustained engagement, and the ease of having and maintaining push and pull messages between users and the system. This level and frequency of engagement were only possible on WhatsApp. WhatsApp’s end-to-end encryption safeguards sensitive health information, addressing key privacy concerns in handling medical data. We did not plan to provide the users with data bundles to use the system; however, our design was also lean in that it did not include any multimedia, which consumes a lot of data for the participants.

**Table 1.** Decision-making discussion with the developers, comparing with Unstructured Supplementary Service Data (USSD).

	WhatsApp	USSD^a^
Pros	It is now the most popular communication platform. There are now low-cost phones that support WhatsApp; the developers advised that most of our participants will have access. The proposed WhatsApp messages are low-cost even to the recipient; therefore, the impact on their affordability will be minimal. WhatsApp supports push services where a service provider can initiate interaction. It is ubiquitous.	It is available on any phone, including those that do not support WhatsApp. It is ubiquitous. It can be reverse billed, so there is no cost to the participant.
Cons	Some phones may not support. WhatsApp but low-cost phones are increasingly being made with WhatsApp support. Participants will always need data to receive and send messages.	It is an old technology that is nearing obsolescence. Costly to the provider, charges 20c per 20 seconds. Does not support a push service. Follow-ups are best done using push messages to eliminate the need for a participant to initiate communication.

a Unstructured Supplementary Service Data.

#### Shesha Workflows

**Figure 3.**
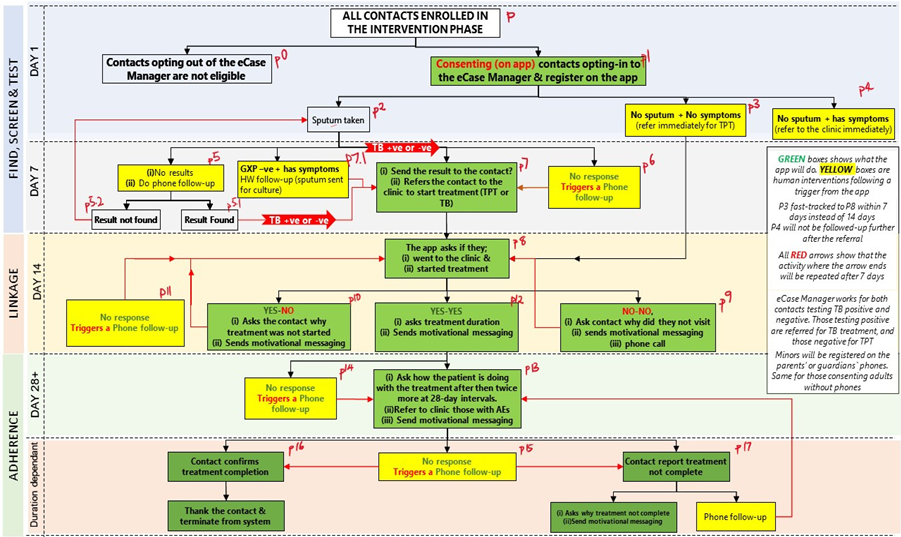
Shesha workflows.

We developed the workflows of Shesha to define the journey contacts take as they navigate through linkage and adherence processes. Figure 3 presents the actual workflows that were conceptualized and eventually translated into Shesha.

#### Key Staff Involved in Implementing Shesha

Table 2 outlines the core personnel responsible for the development and implementation of Shesha. Scientists and researchers conceptualized and designed workflows and user journeys. Research assistants registered household contacts, taught participants how to use the chatbot, and collected sputum samples during household visits. The developers and their technical team translated our design and workflows into a prototype, conducted quality assurance, and built a real-time monitoring dashboard.

While we recognized that Shesha would ideally automate most interactions, there would be situations requiring direct human intervention. We therefore introduced a “Human Case Manager,” mainly responsible for addressing nonresponses to Shesha and pitfalls such as missing results, queries from both contacts and field staff, and any other issues arising from the implementation. The Human Case Manager would step in after receiving a system-triggered alert to intervene. Alerts were included for nonresponses to messages or prompts after 3 unsuccessful automated follow-ups, or if a user had not received test results within a reasonable timeframe.

**Table 2.** Core implementation team.

Role	Responsibilities
Internal	
Scientists and researchers mainly from Aurum and University College London	Led conceptualization and design Developed workflows and user journeys Coordinated integration with other teams Ensured alignment with study objectives and public health goals Oversaw data collection and analysis
RAs^a^ and CHWs^b^	Registered household contacts Taught chatbot usage Captured contact details Screened for TB^c^ symptoms Collected sputum samples Provided initial support and guidance during setup
Human Case Manager	Provided oversight and intervention Managed nonresponses Guided treatment journeys Contacted users who did not respond to automated prompts Ensured user engagement and adherence to treatment protocols Addressed any possible TPT^d^ if contact requested
External	
Developers and technical team	Developed the prototype Conducted quality assurance Built a real-time monitoring dashboard Provided operational manuals and user experience maps Ensured system reliability and effectiveness Delivered training and support for system implementation Continued support and fixing of bugs

a RAs: research assistants.

b CHWs: community health workers.

c TB: tuberculosis.

d TPT: tuberculosis preventive treatment.

### Prototype, Build, and Launch

The building of the prototype started from April 2023 to July 2023, with weekly meetings between researchers and developers to track progress and ensure workflows remained aligned with our original plan. We used Figma (Figma Inc., San Francisco, CA, USA), a collaborative software, to design and visualize user journeys. We compared the workflows with the original Shesha concept (Figure 3) developed. The developers used these visual workflows to translate into a chatbot. For the development, they used TextIt (TextIt, Seattle, WA, USA), a platform that enables the rapid building of user journeys. TextIt was connected to a WhatsApp Business Application Programming Interface through a linkage platform called Turn.io (Turn.io PBC, Denver, CO, USA).

We used local guidelines for TB control and prevention to tailor messages, segmenting them by gender and age to provide personalized engagement. Messages covered key aspects such as symptom monitoring, treatment reminders, and motivational cues, reinforcing the core principles of the HBM by addressing perceived susceptibility, severity, and benefits while providing actionable prompts for care-seeking behavior.

Although we designed Shesha to accommodate multiple users on a single device, WhatsApp natively supports only single-user functionality. Therefore, the developers implemented a structured workaround using the logic architecture of TextIt. Each household contact was assigned a unique set of metadata fields at registration (eg, name, sex, age, screening date, test result), which were stored and used to distinguish individuals sharing the same phone number. TextIt’s flow engine used conditional logic (eg, “if contact_name = X, then routed to Flow A”) to direct messages and automated tasks to the correct user profile. These flows were configured using TextIt’s visual builder, enabling structured branching, user-specific reminder intervals, and status tracking at the individual level. During development, the developers created test scenarios simulating households with 2-5 users on a single device to test conversation threading, state memory, and flow accuracy. The developers also conducted functional quality assurance testing using TestRail (Gurock Software GmbH, Frankfurt, Germany) to verify that messages were correctly triggered per individual, that switching between users could occur without data loss, and that no cross-contamination of prompts occurred. No logic failures were observed in controlled tests, though minor user confusion about navigating between individuals was noted during rollout. This approach has proven stable for typical household sizes (2‐4 users). However, it is still possible that scaling up for larger households may require interface or onboarding enhancements in future iterations.

System monitoring was implemented via a custom dashboard using Amazon Web Services Redshift (Amazon Web Services, Inc., Seattle, WA, USA) and Tableau (Tableau Software, LLC, Seattle, WA, USA) to track flow completion per user, message delivery success, and dropout triggers. The developers managed the Tableau dashboard, which the research team used for daily, interactive tracking of Shesha’s performance by dates and flows. The dashboard displayed raw counts of real-time interactions, which required later data cleaning and deduplication. The research team did not have direct access to modify the dashboard as needed, requiring relaying of information to developers for any change. Nevertheless, the dashboard provided a practical tool for high-level monitoring of key metrics during development.

Full approval for deployment was granted on September 11, 2023, with the service implemented until March 2025.

### Evaluate and Improve

We developed an evaluation plan based on the Reach-Effectiveness Adoption Implementation Maintenance (RE-AIM) framework. The stakeholders had recommended a rigorous evaluation, and, in prior work, we had identified a scarcity of evaluations of mHealth tools.

#### Evaluate

We adopted the RE-AIM framework, which assesses 5 key dimensions: reach, effectiveness, adoption, implementation, and maintenance.

#### Improve

This phase focused on further refinements as we progressed with the implementation. It included addressing bugs and incorporating any user feedback. We continued with regular check-ins with the developers to resolve issues promptly and ensure that the chatbot functioned optimally. The major formal review of Shesha took place in October 2024 through a co-creation workshop held with an external provider to assess Shesha’s human-centeredness, 1 year after enrolling participants. This was made possible due to the additional funding we applied for and secured from a different funder to continue implementing into 2025. We also used these funds to make some changes, mainly an additional language. From the time of its launch, we had been aware of a limitation in having only English as the language on Shesha, so we translated into isiZulu and launched on December 11, 2024, the most widely spoken language in Ekurhuleni. Even with additional funding, budget constraints limited us to only 1 extra language.

### Costing

We calculated Shesha’s development cost using actual invoices paid to the developer, which included capital costs (a 1-time outlay) and recurrent costs. Capital costs were converted into economic costs over 5 years to account for opportunity costs and depreciation, while recurrent costs were not adjusted. The Global Health Costing Consortium acknowledges uncertainties about the useful lives of new technologies [35]. We therefore defaulted to the recommended 5-year useful life assumption, which is used when a useful life is unknown and has been adopted by various projects on mHealth [36-38]. For the discount rate, we used 8%, which reflects the average public interest rate in South Africa, and an exchange rate of 18.33 US dollars.

### Ethical Considerations

The Human Research Ethics Committee (Medical) approved the development and implementation of Shesha as an amendment to the CUT-TB trial under approval number 210107. This amendment revised the project objectives to accommodate the new universal testing and TPT policies. We considered several ethical concerns common when developing Shesha, including informed consent, data privacy, and more complex challenges such as phone sharing. For the actual enrollment of contacts, we first took participants through a consenting process to explain the purpose of the study, potential risks, how their data would be protected, and their rights as participants. In addition to the consent, we provided participants with a study-specific privacy policy, which is required by South Africa’s Protection of Personal Information Act. It explained the storage and processing of personal data. The privacy policy also outlined mechanisms involving multiple users and the risk of unintentional disclosure. We only registered users when both primary and secondary users agreed to the policy. We also added safeguards to Shesha’s design; messages used clear, neutral language, avoiding people’s names or sensitive health terms. At registration, participants were encouraged to use aliases instead of their real names. Test results were delivered only after the user entered a self-created pin, and recruiters trained participants during onboarding on how to use security features such as screen locking to reduce the risk of unauthorized access. Users could opt out at any time via a visible menu option available throughout the platform.

## Results

### Performance

**Figure 4.**
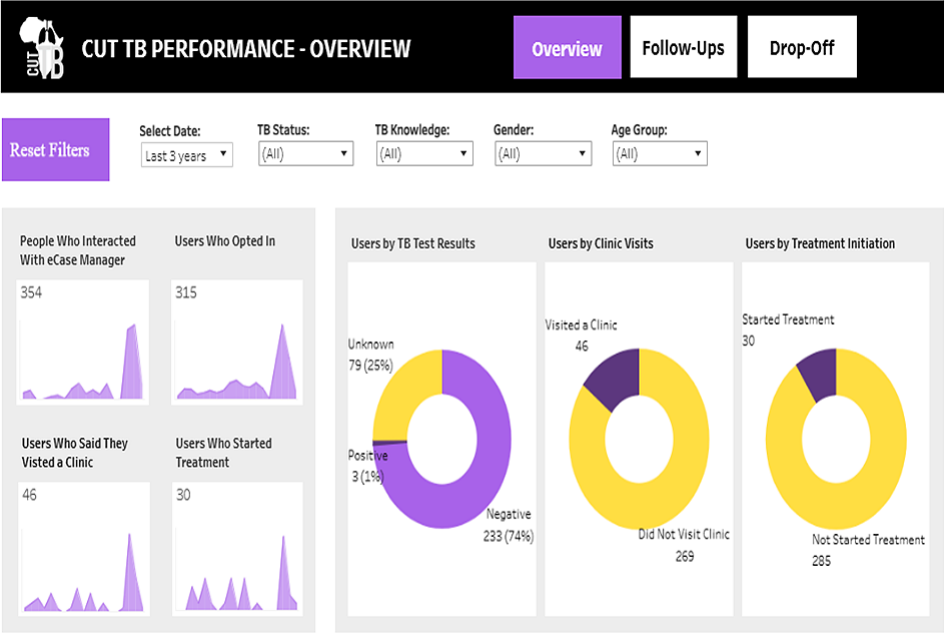
Illustrative tableau dashboard used for monitoring real-time Shesha metrics.

Shesha’s performance was tracked through a Tableau dashboard that presented both counts and percentages at each stage of engagement, allowing the cascade to be pictured as contacts progressed (see Figure 4). The dashboard provided a high-level overview of contact progress from enrollment to treatment, displaying raw counts that facilitated daily monitoring and real-time cascade tracking. However, it required further data cleaning for reliable evaluation. The metrics in Figure 4 show a one-time illustrative snapshot of what the research team would use daily to view performance.

### Summary of Output From the Engagements With Internal and External Stakeholders

A summary of the recommendations from the stakeholder consultation meeting is presented in Table 3. These recommendations addressed existing challenges in the contact tracing process and how we could design the Shesha tool to resolve them. We incorporated most suggestions into the Shesha app, excluding extensive counseling and GPS data collection. Counseling would have required a separate module and additional costs, while GPS was considered unnecessary for information exchange and could have deterred user engagement due to privacy concerns. Instead, we used passive geolocation to allow users to select nearby clinics, assuming most would choose local facilities.

**Table 3.** Summary of suggestions from the internal stakeholder consultation meeting.

Problem identified in the tracing cascade	Recommendations from stakeholders
Notification of results	
Delayed TB^a^ results delay treatment initiation.	Have faster notification to supplement NHLS^b^ SMS
Non-delivery of results or not read if SMS is used	Use WhatsApp to improve the chances of reading messages
Patient follow-up	
No reliable mechanism to show non-responses	Trigger phone follow-ups if no response within 14 days
Limited follow-up may delay initiation.	Prioritize immediate follow-up for high-risk cases
Treatment initiation	
High opt-out rates from the digital system	Collect, analyze, and opt-out the reasons to address the gaps
Patients disengaging before reaching care	
Treatment adherence	
Risk of lower engagement after starting TPT^c^	Send regular adherence messages monthly until completion
Current lack of supportive information	Use a theory-informed behavior change model
Patient support	
Emotional and psychosocial needs not addressed	Integrate counseling sessions into the adherence
Data collection and integrity	
Patient identifiers such as age and gender	Collect basic demographics
Self-reported data may be unreliable	Validate self-report with clinic verifiable data
Limited ability to analyze service gaps geographically	Collect GPS coordinates to enable geospatial analysis
Cost management	
High SMS costs for providers deter sustainability.	Use WhatsApp as a primary platform due to its lower cost and greater functionality

a TB: tuberculosis.

b NHLS: National Health Laboratory Service.

c TPT: tuberculosis preventive treatment.

### Early User-Satisfaction Insights

A user acceptance survey was distributed on Shesha to each user after their initial 30 days of participation for participants enrolled until March 30, 2024. By May 2024, when the survey data were analyzed, only 51 users had been onboarded onto Shesha. A total of 46 surveys were sent to these users because the remaining 5 were internal testers. Of these 46, 12 (26%) participants completed the survey, 30 (65%) users started but did not finish, and 4 (9%) never responded at all. Many of those who stopped after starting only answered up to the third question. Nonetheless, we gained some insights from the users who submitted data. When asked about the perceived safety and privacy of their information, users mainly responded affirmatively. One user commented on the accuracy of the delivery timing or the results.

“I got the results on the exact time frame I was given, and it was a one-on-one session, was able to ask anything I wanted to ask”

However, 1 reported forgetting their onboarding password and could not get results on time.

“I never received any results. I forgot my password”

Users shared that Shesha helped them attend the clinic. Textbox 1 presents respondents’ comments. Overall, participants felt that Shesha’s information improved their understanding of TPT and TB; however, some still believed they did not need to visit the clinic because of negative results.

Textbox 1. **User perceptions about Shesha benefits.**

*“I understand more about TPT”*

*“Everything was clearly explained to me and I understood everything with regards to TB”*

*“I have never went for clinic visits as my results were negative”*

*“It made me feel less anxious in visit the clinic”*

Users held mixed views about how Shesha was motivating them to start TPT after receiving their results. One individual expressed no motivation to begin treatment despite receiving encouraging messages.

“I have never taken any TPT medicine as I have never visited a clinic after getting my test results, as they came back negative.”

However, another user expressed a change in perception regarding starting treatment after receiving messages from the chatbot.

“At first I was scared to take the treatment, but after the charts I understood the importance of taking the medication.”

Regarding the remaining quantitative metrics from the satisfaction survey. A total of 17 responded about the clarity of messaging, and 88% (15/17) reported that the messaging was clear, 94% (16/17) said the menu information was easy to find. Only 15 participants answered the Shesha user friendliness, and 74% (11/15) found the service user-friendly. Overall, for the 15 that continued, their perceptions of Shesha were favorable, with 69% (11/15) rating it as “Good” and 25% (4/11) as “Ok,” and only 1 of the 15 (6%) said it was bad. Importantly, all 15 (100%) indicated they would recommend it to others.

### Changes and Improvements to the Service

#### Resolving Bugs

The user satisfaction survey did not prompt any significant changes to the service. However, some modifications were noted, and bugs encountered were fixed. Textbox 2 provides a summary of the changes that were made to the service as well as the bugs fixed during the process.

Textbox 2. **Summary of issues identified and resolved during quality assurance and early implementation.**

**Description of the issue and bugs**

Workflow breakdowns

*i. Day 10 message sent twice in a multi-user TB-positive flow; Day 14 failed to trigger, and the system froze*.

*ii. Days 14, 20, and 28 did not trigger automatically; manual start led to incorrect flow assignment*.

*iii. Users who intended to visit the clinic did not receive the required 7-day follow-up message*.

*iv. Day 7 was skipped entirely in the user journey; the system jumped to Day 10*.

*v. Flow became unresponsive after fast-forwarding a user to Day 3 post-onboarding*.

*vi. “Add more people” and “Done” buttons did not appear after registering the first user*.

*vii. After adding 6 users, the system sent inbox messages in the wrong sequence (group then random individual*).

*viii. “Stop messages” command looped back to the previous step instead of terminating communication*.

**Missing or incomplete prompts**

*i. Day 14 question on medication collection was omitted, affecting follow-up*.

*ii. Completion message after onboarding lacked a prompt linking users to TB education via the Menu*.

*iii. Final message on Day 3 lacked instructions for accessing results in the secure inbox*.

*iv. Responding “No” in the Day 20 symptom check did not trigger the expected debug message and email alert*.

*v. Automated email alerts omitted user identifiers (eg, phone number, participant ID) or contained incorrect usernames*.

*vi. Selecting “Other” as a clinic option failed to trigger a free-text input prompt; flow skipped ahead*.

**Content and messaging inconsistencies**

*viii. Day 20 (side effects) and Day 28 (adherence) messages were sent at the same time, which was confusing*.

*ix. The WhatsApp message differed from the approved Figma design, raising concerns about consistency*.

*x. Steps 3 and 6 in onboarding gave inconsistent instructions from the mock-up*.

### Summary of Human-Centered Design Workshop Changes and Recommendations

**Figure 5.**
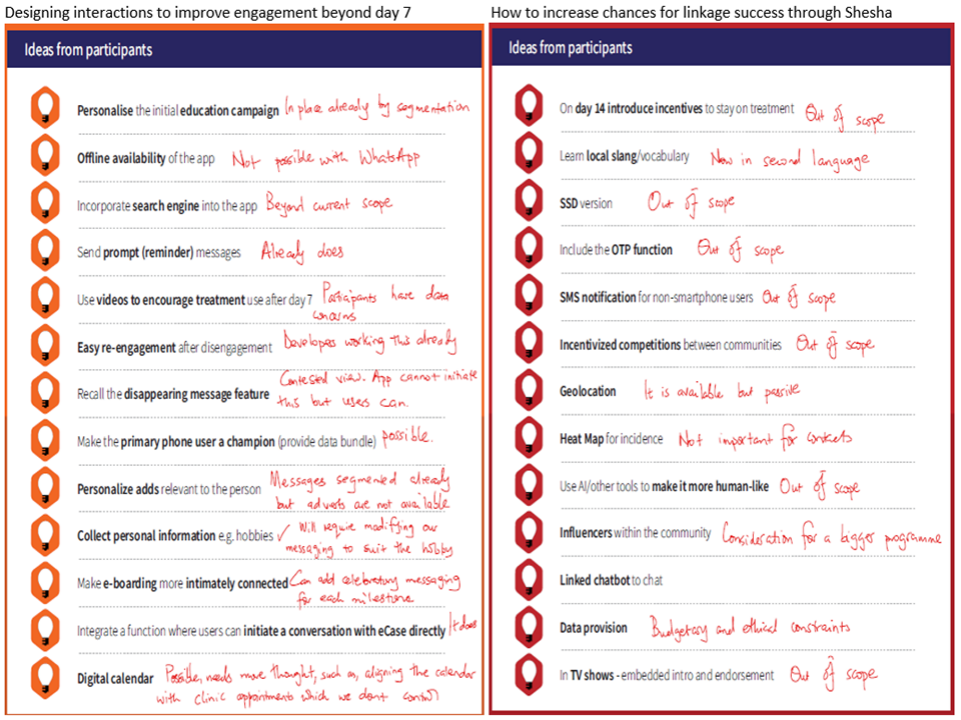
Recommendations from the codesign workshop.

A workshop was held 1 year after the launch of Shesha where delegates suggested ways to improve the chatbot and also recommended alternative prototype models. A total of 2 key questions arose: (1) how to design interactions for engagement beyond day 7, and (2) how to increase success in linkage goals. We annotated responses to each recommendation; most were already included or outside scope due to budget (see Figure 5). A significant change was renaming the chatbot from being called eCase Manager to its new name Shesha. The new name was from IsiZulu, the most spoken local language in South Africa, which means “hurry.” The name aligned better with the its purpose to link people faster and also removed the mislabeling of people as “cases.” Changing the name to appear as Shesha on WhatsApp was, however, complex due to Meta Business Suite requiring updates like changing the display name on the website of the research team and a subsequent Meta review. The project came to an end before finalizing the official name change; however, we plan to rename again if we secure more funding to continue implementation. In the field, however, the staff had already started referring to the service as Shesha and introducing it as such to the participants.

### Multiple User Journeys

**Figure 6.**
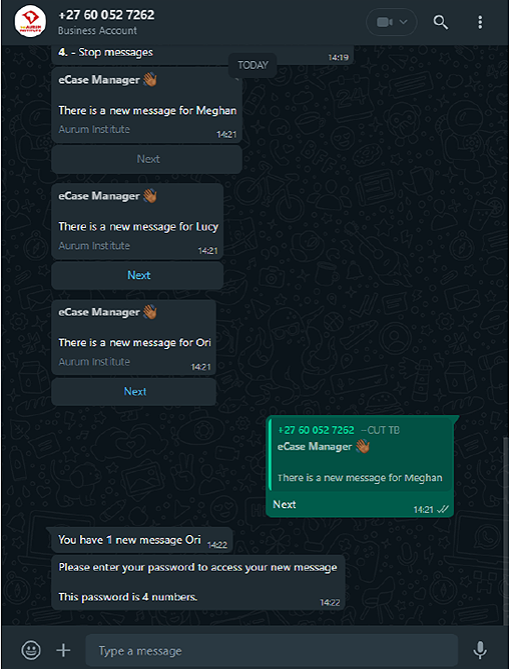
Example of a multiuser journey.

Multiple user journeys were chosen to avoid excluding household members without phones; however, these were difficult to implement and required workarounds. Having multiple users on 1 number complicated workflows because the system needed to identify the user in the session to link responses correctly. For example, if a household had multiple users and messages were sent in the morning (staggered by 1 h from 9 AM), the reply only accepted the most recent message. As shown in Figure 6, if messages are sent to “Meghan,” “Lucy,” and “Ori” and “Meghan” replies, Shesha would recognize the latest message from “Ori.” In multiple-user households, messages were designed to be sent an hour apart, allowing the recipient to respond. In addition, to ensure proper workflow, each user received a participant ID at onboarding and chose a name or alias to use for identifying themselves in their responses.

### Evaluation and the Cost of Developing Shesha

The indicators for the planned process evaluation using the RE-AIM framework are shown in Table 4. These indicators cover the implementation science outcomes with the aim of understanding the real-world factors that would influence success or failure of the app. The evaluation results are, however beyond the current scope, which is only focusing on documenting the development process.

**Table 4.** RE-AIM evaluation indicators.

RE-AIM^a^ domain	Definition of indicators to be measured
Reach	Proportion of household contacts who were found, screened for eligibility, and successfully registered on Shesha
Effectiveness	Proportion of Shesha users who attended clinic, started TB^b^ or TPT^c^, completed treatment
Adoption	Proportion of Shesha users who acted on (opened, read, or responded) to milestone messages (Days 3, 5, 7, 14)
Implementation	*Internal fidelity*: whether Shesha executed design logic correctly (queuing contact, delivering messages, sending reminders) *External fidelity*: whether staff followed onboarding steps *Feasibility*: whether users had the conditions to engage (phone access, literacy)
Acceptability	User perceptions of usefulness, ease of use, motivation, and barriers, structured by UTAUT^d^ constructs
Maintenance	Continued engagement after Day 28, including responses to monthly follow-ups

a RE-AIM: Reach Effectiveness Adoption Implementation Maintenance.

b TB: tuberculosis.

c TPT: tuberculosis preventive treatment

d UTAUT: Unified Theory of Acceptance and Use of Technology.

In addition to the process evaluation, Table 5 shows the total accounting and economic costs of developing and supporting Shesha. Using a 5-year lifespan, an 8% discount rate consistent with South Africa’s public rate, and an exchange rate of R18.33 per USD, the 19-month economic cost was US $142,754. The economic cost of development was US $16,388 annually over 5 years, with yearly support costs totaling US $73,773. In total, Shesha would cost US $90,160 per year.

**Table 5.** The cost of developing Shesha.

Item	Cost category	Accounting cost (USD)^a^	Equivalent monthly cost (USD)	Equivalent annual cost (USD)	19-Month cost (USD)
Development costs	Capital	56,593	1166	13,988	22,147
Translations	Capital	9709	200	2400	3800
Subtotal capital	Capital	66,302	1366	16,388	25,947
Annual support	Recurrent	116,807	6148	73,773	116,807
Total economic cost	All	183,109	7513	90,160	142,754

a US dollars.

## Discussion

### Principal Findings

This paper describes the development of Shesha, a WhatsApp-based chatbot designed to support household contacts of people being treated for TB in their linkage to care in South Africa. We applied a design thinking process, using the HBM to structure the user journey. Published accounts of mHealth tool development are scarce in South Africa, and to our knowledge, this is the first peer-reviewed paper documenting such a process on TB contact tracing in South Africa. Only 1 other local study, by Mathenjwa et al [39], reports on the development of a TB and HIV tool, but the tool is more broadly used, unlike Shesha that focuses on contact tracing. Our work, therefore, addresses a significant gap in public health–oriented design literature in this setting.

Using design thinking helped translate technically demanding workflows into a clear structure, making development feel more manageable despite the complexity of the tool. Several studies have shown that design thinking enhances the development of digital health tools [26,40]. Except for the delayed user satisfaction survey, in Shesha, we largely followed the framework’s steps. In retrospect, a more flexible application of the framework, as suggested by others [41], might have enabled more structured stakeholder engagement, including workshops and regular feedback loops. Despite this, the design thinking approach added significant value and should remain central to future tool development efforts.

Collaborating with skilled developers was essential for Shesha’s progress. We partnered with a team experienced in public health to ensure technical decisions aligned with program goals and real-world implementation. Usually, public health teams identify problems, and developers build the architecture; imbalances between and asymmetries in interest can hinder progress. To prevent this, we formalized the initial partnership with early agreements on intellectual property, code ownership, change procedures, and post-launch responsibilities, which fostered a productive working relationship. Therefore, partnering with developers should be viewed not just as a client-vendor relationship, but as a genuine collaboration focused on shared benefits.

Dashboards are essential for managing chatbot interventions, which generate large volumes of data across different flows and time points. In Shesha, the dashboard was built and managed externally by the developers. It provided real-time visibility and supported day-to-day monitoring, but this arrangement limited our ability to make changes quickly when needed. Although the dashboard included basic validation checks, it used raw data and could not produce outcome-level indicators without additional data cleaning. A similar approach was used in the TBCheck project in South Africa, where dashboards supported implementation, but final outcomes were confirmed through laboratory records Rich [42]. More work and skill capacitation are needed to ensure that public health teams can exert greater control over the design, management, and use of dashboards. We agree with Rossi et al [43] in their argument that dashboard development should follow a dedicated design thinking process, which can strengthen their role as core tools for monitoring and guiding digital health interventions.

WhatsApp was selected because it offered the best balance between functionality, engagement, and cost. SMS and USSD were considered, but resource constraints limited the use of multiple platforms. More importantly, these platforms were not well suited to the kind of 2-way, conversational engagement that Shesha required. WhatsApp allowed us to deliver motivational messages in a familiar, chat-based format that aligned with how people already communicate and was more likely to sustain interest. At the same time, we were aware of the access challenges this choice might present. Although over 75% of South Africans use smartphones to access the internet [23], TB-affected households are disproportionately affected by poverty and may struggle to afford data or maintain devices [44-46]. A South African study recommended SMS over WhatsApp for this reason [47]. However, the study targeted recently circumcised men, a group with high perceived risk and strong motivation to engage. Our context was different. Shesha targeted generally healthy household contacts, being asked to start preventive treatment for a disease they did not see as an immediate threat. According to the HBM, perceived risk and susceptibility influence engagement, and in our case, ease of use and familiarity with the platform were probably more significant than broad accessibility alone. Platform choice, therefore, should not be based only on cost or reach. Even inexpensive options can fail if they impose all the burden on implementers who cannot or will not bear the costs. The decision must consider behavioral, social, financial, and technical factors specific to the context.

Regarding ethics and information privacy, especially for shared devices, privacy can never be fully guaranteed because messages are not invisible to all users on the device. Our methods for providing PINs to access sensitive information become obsolete once the message is received and left undeleted. Ethical issues in WhatsApp use remain contentious despite safeguards like encryption. One review of health studies highlighted gaps in privacy, emphasizing the need for minimal data collection, anonymization, and transparent risk communication [48]. In our study, we implemented these safeguards: using aliases, providing detailed consent, and adding extra privacy policies. However, we recognized that ultimate control over security depends on the platform provider, and protections are only as adequate as those put in place by WhatsApp itself. These issues extend beyond individual studies like ours and need to be addressed at a governance and regulation level.

Finally, while Shesha was built using preprogrammed logic trees, advances in artificial intelligence (AI) now offer new opportunities for dynamic, adaptive conversation flows. At the time of development, AI integration was not widely available. However, the recent rapid evolution of AI in chatbot platforms means that tools like Shesha could, in the future, work more intelligently to respond to user inputs.

### Limitations and Lessons Learned

The development of Shesha was not without its challenges. A commonly expected step in digital health development is to conduct pre-intervention surveys or user consultations to guide design, which we could not do in our study, as described in the *Methods* section. This absence may have delayed or limited the discovery of usability issues that only surfaced during actual use. For instance, although prior studies were helpful, they may not have fully captured the profile of this novel population, resulting in gaps in areas such as the timing of messages for multiple-user households or participants’ preferences for privacy features beyond PINs, which we used in Shesha. Some challenges may also not have been realistically identifiable before use, as users had no prior exposure to TPT or patient-facing digital tools. This makes the absence of a predesign survey both a limitation of our approach and a reflection of the development context. However, it was not a dealbreaker; instead, it offers a lesson for future developers. Predesign consultations remain a standard part of digital health projects and should be undertaken wherever possible. In settings like ours, where valuable input may not be available until users experience the tool directly, implementers must anticipate the risks of postponing engagement and plan for structured feedback after launch, along with the ability to implement timely updates.

Budget constraints affected both the pace and flexibility of development. Developer time was tightly allocated to the initial build, making it difficult to accommodate changes once development was underway without securing additional funds. This limitation contributed to the initial launch of the tool in English only, which may have reduced accessibility for non-English-speaking users. Although additional funding later allowed for translation into isiZulu, the delay highlights how resource constraints can affect inclusivity. Our costing exercise also showed that development and support costs can be high relative to the scale of early implementation, which may discourage smaller organizations from pursuing similar tools. As funding streams become more limited, future mHealth efforts will need to present strong evaluation data and transparent development documentation to build a credible investment case and secure sustainable support.

### Conclusion

The development of Shesha offers valuable insights for future mHealth design and implementation in resource-limited settings. Structured design methods like design thinking remain useful and should inform development efforts. However, flexibility is crucial, as real-world circumstances may require deviation from standard sequences or planned processes. Equity considerations should be integrated early in technical decisions, even if they add complexity to implementation. This involves ensuring access for various user groups and accommodating different device usage patterns. Effective collaboration between public health teams and developers is essential to aligning technical solutions with program aims. Partnerships based on shared planning and mutual understanding can minimize friction and support successful delivery. Platform selection, budgeting, and ethical safeguards need to be considered within the specific context. Tools should aim to balance reach, functionality, sustainability, and privacy, especially when engaging populations with limited resources or digital access. Lastly, documenting and sharing the development process, including challenges and trade-offs, is just as important as reporting outcomes. Transparent documentation strengthens the evidence base for mHealth, encourages learning across projects, and helps build a stronger case for sustained investment in digital health innovation.

## Abbreviations

CHW: community health worker
CUT-TB: Community and Universal Testing for Tuberculosis among Contacts
HBM: Health Belief Model
mHealth: mobile health
RE-AIM: Reach Effectiveness Adoption Implementation Maintenance
TB: tuberculosis
TPT: tuberculosis preventive treatment
USSD: Unstructured Supplementary Service Data

## References

[1] Brinkel J, Krämer A, Krumkamp R, May J, Fobil J (2014). Mobile phone-based mHealth approaches for public health surveillance in sub-Saharan Africa: a systematic review. Int J Environ Res Public Health.

[2] Van der Pol N, Ntinga X, Mkhize M, van Heerden A (2022). A scoping review of mHealth use in South Africa. Dev South Afr.

[3] Research and Training in Health and Development (RTHD) (2019). Review of mHealth solutions for TB in South Africa. https://tbthinktank.org/wp-content/uploads/2022/08/2019-Policy-Brief-mHealth-for-TB-in-South-Africa.pdf.

[4] Mudzengi DL, Chomutare H, Nagudi J (2024). Using mHealth technologies for case finding in tuberculosis and other infectious diseases in Africa: systematic review. JMIR Mhealth Uhealth.

[5] Perrin Franck C, Geissbuhler A, Lovis C (2023). “To err is evolution”: we need the implementation report to learn. JMIR Med Inform.

[6] Iribarren SJ, Beck SL, Pearce PF, Chirico C, Etchevarria M, Rubinstein F (2014). mHealth intervention development to support patients with active tuberculosis. J Mob Technol Med.

[7] Katende KK, Amiyo MR, Nabukeera S (2022). Design, development, and testing of a voice-text mobile health application to support tuberculosis medication adherence in Uganda. PLoS ONE.

[8] (2024). Global tuberculosis report 2024. World Health Organisation.

[9] Fox GJ, Barry SE, Britton WJ, Marks GB (2013). Contact investigation for tuberculosis: a systematic review and meta-analysis. Eur Respir J.

[10] Müller J, Kretzschmar M (2021). Contact tracing—old models and new challenges. Infect Dis Model.

[11] Baluku JB, Nabwana M, Winters M, Bongomin F (2022). Tuberculosis contact tracing yield and associated factors in Uganda. BMC Pulm Med.

[12] Guy D, Kodjamanova P, Woldmann L (2025). Contact tracing strategies for infectious diseases: a systematic literature review. PLOS Glob Public Health.

[13] Velen K, Lewis JJ, Charalambous S (2016). Household HIV testing uptake among contacts of TB patients in South Africa. PLoS ONE.

[14] Armstrong-Hough M, Turimumahoro P, Meyer AJ (2017). Drop-out from the tuberculosis contact investigation cascade in a routine public health setting in urban Uganda: a prospective, multi-center study. PLoS ONE.

[15] (2023). National guidelines on the treatment of tuberculosis infection. National Department of Health South Africa.

[16] (2024). South africa national TB strategic plan: 2023-2028. south african national TB programme. National Department of Health South Africa.

[17] Moyo M, Ntshiqa T, Hamada Y (2025). Community and universal testing for TB among close contacts of microbiologically confirmed pulmonary TB patients in two high TB burden countries: a protocol for a pragmatic cluster-randomised control trial. Trials.

[18] (2022). Ekurhuleni municipality integrated development plan—2022/23–2026/27. Ekurhuleni Municipality.

[19] Mukora R, Thompson RR, Hippner P (2023). Human resource time commitments and associated costs of Community Caregiver outreach team operations in South Africa. PLoS ONE.

[20] (2022). Annual report 2021/22. Ekurhuleni Municipality.

[21] (2024). Curable TB claims 1.5 million lives per year. Gauteng Department of Health.

[22] Kufa-Chakezha T, Shangase N, S B, Cutler E, Aitken S, Cheyip M (2023). The 2022 antenatal HIV sentinel survey: key findings. Public Health Bulletin South Africa.

[23] Miyajima K (2022). Mobile phone ownership and welfare: evidence from South Africa’s household survey. World Dev.

[24] Moyo S, Ismail F, Mkhondo N (2023). Healthcare seeking patterns for TB symptoms: findings from the first national TB prevalence survey of South Africa, 2017-2019. PLoS ONE.

[25] Dam RF (2024). Design thinking: Interaction Design Foundation.

[26] Fähnrich C, Denecke K, Adeoye OO (2015). Surveillance and outbreak response management system (SORMAS) to support the control of the Ebola virus disease outbreak in West Africa. Euro Surveill.

[27] Grainger C (2020). A software for disease surveillance and outbreak response-insights from implementing SORMAS in nigeria and ghana. Federal Ministry for Economic Cooperation and Development (BMZ).

[28] MacPherson M, Merry K, Locke S, Jung M (2022). Developing mobile health interventions with implementation in mind: application of the multiphase optimization strategy (MOST) preparation phase to diabetes prevention programming. JMIR Form Res.

[29] Thind D, Charalambous S, Tongman A, Churchyard G, Grant AD (2012). An evaluation of “Ribolola”: a household tuberculosis contact tracing programme in North West Province, South Africa. Int J Tuberc Lung Dis.

[30] Rosenstock IM, Strecher VJ, Becker MH (1988). Social learning theory and the Health Belief Model. Health Educ Q.

[31] Praekelt.org. TB healthcheck puts tuberculosis self-screening in everyone’s hands ahead of World TB Day.

[32] Barron P, Peter J, LeFevre AE (2018). Mobile health messaging service and helpdesk for South African mothers (MomConnect): history, successes and challenges. BMJ Glob Health.

[33] Cowling N Number of internet users in South Africa from 2013 to 2024: Statista.

[34] Cowling N Leading social media platforms in South Africa 2023: Statista.

[35] DeCormier Plosky W, Bollinger LA, Alexander L (2019). Developing the global health cost consortium unit cost study repository for HIV and TB: methodology and lessons learned. Afr J AIDS Res.

[36] Unsworth M, Fabens I, Setswe G (2025). Expanding two-way texting for post-operative follow-up: a cost analysis of the implementation and scale-up in routine voluntary medical male circumcision settings in South Africa. PLOS Glob Public Health.

[37] Chen Y, Ronen K, Matemo D (2020). An interactive text messaging intervention to improve adherence to option B+ prevention of mother-to-child HIV transmission in Kenya: cost analysis. JMIR Mhealth Uhealth.

[38] Khan ZA, Kidholm K, Pedersen SA (2024). Developing a program costs checklist of digital health interventions: a scoping review and empirical case study. Pharmacoeconomics.

[39] Mathenjwa T, Adeagbo O, Zuma T (2020). Development and acceptability of a tablet-based app to support men to link to HIV care: mixed methods approach. JMIR Mhealth Uhealth.

[40] Johansson‐Sköldberg U, Woodilla J, Çetinkaya M (2013). Design thinking: past, present and possible futures. Creat Innov Manage.

[41] Altman M, Huang TTK, Breland JY (2018). Design thinking in health care. Prev Chronic Dis.

[42] Rich K, Burger R, Goldberg D, Moultrie H, Rieger M (2025). Is it possible to encourage TB testing and detect missing TB cases via community-level promotion of a self-screening mobile application? Quasi-experimental evidence from South Africa. BMJ Health Care Inform.

[43] Rossi FS, Adams MCB, Aarons G, McGovern MP (2025). From glitter to gold: recommendations for effective dashboards from design through sustainment. Implement Sci.

[44] Foster N, Vassall A, Cleary S, Cunnama L, Churchyard G, Sinanovic E (2015). The economic burden of TB diagnosis and treatment in South Africa. Soc Sci Med.

[45] Erlinger S, Stracker N, Hanrahan C (2019). Tuberculosis patients with higher levels of poverty face equal or greater costs of illness. Int J Tuberc Lung Dis.

[46] National TB patient cost survey findings. Knowledge Hub.

[47] Fabens I, Makhele C, Igaba NK (2024). WhatsApp versus SMS for 2-way, text-based follow-up after voluntary medical male circumcision in South Africa: exploration of messaging platform choice. JMIR Form Res.

[48] Manji K, Hanefeld J, Vearey J, Walls H, de Gruchy T (2021). Using WhatsApp messenger for health systems research: a scoping review of available literature. Health Policy Plan.

